# Mean Attitude Score Regarding Dental Floss among Dentists in a District of Nepal: A Descriptive Cross-sectional Study

**DOI:** 10.31729/jnma.6286

**Published:** 2022-01-31

**Authors:** Shristi Kafle, Erika Shrestha, Rajib Chaulagain, Bashu Raj Pandey

**Affiliations:** 1 Department of Periodontoiogy and Oral Impiantoiogy, Chitwan Medical College and Teaching Hospital, Chitwan, Nepal; 2 Department of Oral Biology, Chitwan Medical College and Teaching Hospital, Chitwan, Nepal; 3 Department of Orthodontics, Chitwan Medical College and Teaching Hospital, Chitwan, Nepal

**Keywords:** *attitude*, *dental floss*, *dentists*, *knowledge*, *oral hygiene*

## Abstract

**Introduction:**

Oral health is an inherent part of the overall individual's general health. Good oral hygiene practices are widely considered essential in maintaining good oral health and flossing has long been considered an indispensable part of an effective oral hygiene routine. This study aimed to find out the mean attitude score regarding dental floss among dentists.

**Methods:**

A descriptive cross-sectional study was conducted among 142 dentists practicing in a district of Nepal from February 2020 to April 2020. Convenience sampling was performed to reach the sample size of 142. Data was collected through both self-administered and online questionnaires sent through google forms after ethical approval from the Institutional Review Committee (Reference number: IRC/076/077-131). Data analysis was done by using Statistical Package for Social Sciences version 20.0 software. Point estimate at 95% confidence interval was calculated along with mean, standard deviation, frequency, and proportion.

**Results:**

The mean attitude score regarding dental floss among dentists was 53.77±4.01 (53.11-54.42 at 95% Confidence Interval). Positive attitude was observed in the majority of dentists 120 (84.5%). Twenty-two (15.5%) were found to have a neutral attitude and none of them possessed a negative attitude towards dental floss similar to other studies.

**Conclusions:**

Based on results of this study, the mean attitude score regarding dental floss among dentists in our study is similar to the findings of other studies. The majority of dentists were found to have a positive attitude towards dental floss.

## INTRODUCTION

Good oral hygiene practices are widely considered essential for maintaining good oral health. Dental flossing has long been considered an indispensable part of an effective oral hygiene regimen. Dental floss available in variety of different sizes and configurations is considered as the "gold standard" of interdental care.^1^ It has also been found that flossing and brushing of interdental spaces improves dental and periodontal health which might reduce the risk for new cardiovascular events among patients with coronary heart disease and hence, its importance has to be highlighted.^2^

Dentists can play a prime role in emphasizing use of dental floss, also educating the general population and the community during community health outreach programs as they encounter various groups of people with different oral diseases during their practices.

This study aimed to find out the mean attitude score regarding dental floss among dentists.

## METHODS

A descriptive cross-sectional study was conducted in Chitwan from February 2020 to April 2020. Before commencing this study, ethical clearance with an ethical approval number as CMC-IRC/076/077-131 was obtained from the Institutional Review Committee (IRC), Chitwan Medical College and Teaching Hospital (CMCTH).

Inclusion criteria were dentists of both genders of any age practicing in Chitwan both in private and government sectors as well as teaching institutions only those who gave consent. Dentists were taken into consideration as they have a direct role in oral health care and promotion, preventive information dissemination and thus it is necessary that their oral health-related knowledge be sound so that the whole community also gets benefited eventually. Participation was voluntary and utmost confidentiality and personal identity of all the participants were assured.

Convenience sampling method was used. Sample size was calculated using the formula,

n = Z^2^ × σ^2^ / e^2^

  = (1.96)^2^ × 2.25^2^/ (0.4)^2^

  = 122

Where,

n= minimum required sample size
Z= 1.96 at 95% Confidence Interval (CI)
σ= standard deviation calculated considering upper and lower margin as 54 and 45 at 95% Confidence Interval respectively (educated guess)
e= lower margin of error taken as 0.4

The minimum sample size required is 122 participants. Considering 10% non-response rate of study participants, the sample size calculated is 135. However, samples of 142 participants were obtained. Data collection was carried out using an online questionnaire. Semi-structured questionnaires designed in English language were developed after reviewing previously published related studies.^3^ The questionnaire was pretested in a pilot phase among 30 dentists to ensure the quality and face validity of the data collection tool. After analyzing the pretested data, 18 closed-ended questions were finalized, which fulfilled the objective of the study. Based on the pretesting results, few modifications were made in the questionnaire after seeking the suggestions from subject experts. Due to a lockdown generated due to pandemic outbreak of coronavirus disease 2019 (COVID-19) implied by the government of Nepal, we were obliged to collect our data by creating and sending links of online questionnaires prepared using Google forms. Electronically distributed questionnaire was selected in order to reach out to all dentists as possible.

Questionnaire was divided into two sections. The first section or preliminary section consisted of sociodemographic details and professional aspects of participants.

The second section contained 14 questions related to the attitude of the dentists toward the use of dental floss which was measured by a 5-point Likert scale. The rating scale was measured as: positive statement with choices strongly agree, agree, neither agree nor disagree, disagree, and strongly disagree and scores 5, 4, 3, 2, and 1, respectively in order to indicate the degree of agreement with regards to essentiality of dental floss, compliance in the use of dental floss, awareness and education in relation to dental floss. The scores varied from 0 to 70, and all individual answers were summed up for total scores and calculated for mean. The scores were classified into three levels as positive attitude, neutral attitude, and negative attitude with respective scoring of 50-70, 35-49 and 0-34.

All returned filled questionnaires were checked for completeness, inconsistencies of response manually; coded, entered into Microsoft Excel version 2018 and transformed to Statistical Package for Social Sciences (SPSS) version 20.0 software for statistical analysis. All answers were treated with utmost confidentiality. Data were analyzed using appropriate descriptive statistics. Point estimate at 95% confidence interval was calculated along with mean, standard deviation, frequency, and proportion.

## RESULTS

The mean attitude score regarding dental floss among dentists was 53.77±4.01 (53.11-54.42 at 95% Confidence Interval). Positive attitude was observed in the majority of dentists 120 (84.5%). Twenty-two (15.5%) were found to have a neutral attitude and none of them possessed a negative attitude towards dental floss similar to other studies (Table 1).

**Table 1:** Showing attitude score and attitude level of dentists towards dental floss.

Attitude	Minimum Score	Maximum Score	Mean
	43	62	53.77 ± 4.01
Attitude level	Positive n (%)	Neutral n (%)	Negative n (%)
	120 (84.5)	22 (15.5)	-

Distribution of study respondents according to their attitude towards application of dental floss is tabulated below. While considering their attitude, 84 (59.2%) strongly agreed dental floss as an essential oral hygiene aid along with the toothbrush. Fifty-six (39.4%) disagreed that toothbrush and toothpaste are enough to remove plaque and debris. Sixty-one (43.0%) of the respondents strongly agreed that routine dental flossing is necessary and 71 (50.0%) agreed that dental floss has a significant role in the maintenance of good periodontal health (Table 2).

**Table 2:** Participants' attitude regarding dental floss (n=142).

Questions	Responses	n (%)
Toothbrush and toothpaste are enough to remove plaque and debris	a. Strongly agree	4 (2.8)
	b. Agree	52 (36.6)
	c. Neutral	18 (12.7)
	d. Disagree	56 (39.4)
	e. Strongly disagree	12 (8.5)
Dental floss is an essential oral hygiene aid along with the toothbrush	a. Strongly agree	84 (59.2)
	b. Agree	49 (34.5)
	c. Neutral	9 (6.3)
Dental floss has a significant role in the maintenance of good periodontal health	a. Strongly agree	58 (40.8)
	b. Agree	71 (50.0)
	c. Neutral	13 (9.2)
Routine dental flossing is necessary	a. Strongly agree	61 (43.0)
	b. Agree	57 (40.1)
	c. Neutral	17 (12.0)
	d. Disagree	7 (4.9)
Lack of compliance in using dental floss	a. Strongly agree	25 (17.6)
	b. Agree	93 (65.5)
	c. Neutral	17 (12.0)
	d. Agree	6 (4.2)
	e. Strongly disagree	1 (0.7)
Dental floss is freely available	a. Strongly agree	23 (16.2)
	b. Agree	52 (36.6)
	c. Neutral	22 (15.5)
	d. Disagree	26 (18.3)
	e. Strongly disagree	19 (13.4)
Dental flossing is a time-consuming procedure	a. Strongly agree b. Agree c. Neutral d. Disagree e. Strongly disagree	6 (4.2) 41 (28.9) 31 (21.8) 51 (35.9) 13 (9.2)
Dental floss is expensive	a. Strongly agree b. Agree c. Neutral d. Disagree e. Strongly disagree	9 (6.3) 33 (23.2) 52 (36.7) 37 (26.0) 11 (7.8)
Dental floss is not as well marketed as other oral hygiene aids in Nepal	a. Strongly agree b. Agree c. Neutral d. Disagree e. Strongly disagree	32 (22.5) 85 (59.9) 15 (10.6) 9 (6.3) 1 (0.7)
Lack of awareness regarding dental floss among people in Nepal	a. Strongly agree b. Agree c. Neutral d. Disagree	50 (35.2) 87 (61.3) 2 (1.4) 3 (2.1)
Dentist should create awareness and motivate people to use dental floss regularly	a. Strongly agree b. Agree c. Neutral	87 (61.3) 53 (37.3) 2 (1.4)
Dental flossing should be taught at the school level, to be incorporated as a routine oral hygiene measure	a. Strongly agree b. Agree c. Neutral	73 (51.4) 68 (47.9) 1 (0.7)
Dental floss is not given adequate importance in undergraduate and postgraduate dental education	a. Strongly agree b. Agree c. Neutral d. Disagree e. Strongly disagree	16 (11.3) 53 (37.3) 26 (18.3) 38 (26.8) 9 (6.3)
Greater emphasis is needed on dental flossing training/education in the dental curriculum	a. Strongly agree b. Agree c. Neutral d. Strongly disagree	54 (38) 81 (57) 6 (4.3) 1 (0.7)

Questionnaires were distributed to total 180 dentists working at private and government sectors as well as in teaching institutions in Chitwan; out of which only 142 dentists responded. Among them, 107 were of undergraduate level and the remaining 35 were specialists from various sub branches of dentistry. Likewise, other professional aspects of the respondents were summarized (Table 3).

**Table 3:** Professional characteristics of the study participants (n=142).

Professional characteristics	n (%)
**Qualifications**	
BDS	107 (75.3)
MDS	35 (24.7)
**Area of practice**	
Government Hospital	11 (7.8)
Private Practice	44 (31.0)
Teaching Institution	87 (61.2)
**Years of practice**	
1-3 years	86 (60.6)
4-6 years	9 (6.3)
7-10 years	19 (13.4)
More than 10 years	28 (19.7)

**Figure 1:**
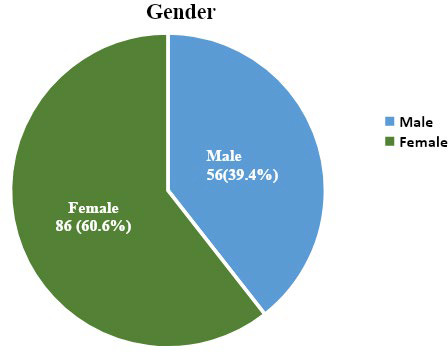
Gender wise distribution of the study participants.

Minimum and maximum age of the respondents were 23 and 65 respectively. The mean age of the study respondents was 29.04±6.07. The gender wise distribution of study respondents is as given below (Figure 1).

## DISCUSSION

It is of paramount importance to prevent dental problems before they start. The easiest way is to practice daily brushing and flossing that in turn will reduce oral diseases.^4^

Dental floss is regarded as a key element in proper oral hygiene as early as the Prehistoric period wherein horsehair was used as a floss. An American dentist, also the Father of oral hygiene Dr. Levi Spear Parmly had been credited for concocting the cutting edge dental floss utilizing waxed silken thread in 1815. He suggested flossing with a bit of silk string, through the interstices of the teeth, between their necks and the curves of the gum, to remove that chafing matter which cannot be evacuated by brush and which is the genuine source of infection. Indeed a book named "A Practical Guide to the Management of Teeth" was published by him emphasizing the importance of brushing and flossing daily.^5^

The silk was afterward supplanted by nylon as the most fabric in floss due to rising costs of silk amid World War II in 1940s and its inclination to shred. This improvement is credited to Dr. Charles Bass, who is well exceptional for making floss a fundamental portion of daily oral hygiene practices. In spite of the fact that the most punctual adaptations of floss were comparative to what is found nowadays, there have been very a number of progressions within the plan to assist streamline the method and make a more comfortable experience.^6^

Tooth brushing and dental flossing are indispensable modalities of self-care practices for prevention of dental caries and periodontal disease. Increased awareness and proper oral health practices are important for better oral health. But the statistics throughout the world is not encouraging despite the recommendation of dental floss along with regular tooth brushing.^7,8^

Keeping this in mind and also the expected role to be played by the dentists, we felt a need for assessing the attitude with regards to dental floss among them. It is also important to identify the deficiencies and barriers among the dentists if any which would be helpful to plan corrective measures.

Due to the educational level and the professional role of the dentists in suggesting effective oral hygiene aids for maintenance of good oral health, they are expected to have positive behavior towards oral health and its diseases which is evident from the baseline data of our study where 120 (84.5 %) had positive attitude towards dental floss.

Ninety-three (65.5 %) of respondents in our study agreed on a fact that there is lack of patient compliance in using dental floss. This could be recognized as one of the factors for less prescription of dental floss. As early as 1995, Lang, et al. came up with similar findings that many people had difficulty accomplishing this with traditional dental floss. It had also been documented that about 30% of the adult population use floss, and even fewer (22%) use it correctly.^9^

Additionally, Shibly, et al. also reported that when given a preference, most patients choose an alternative device over manual floss.^10^ After analyzing the findings of a study carried out in Zambia, the author acknowledges the reason for not flossing may be lack of awareness on the gadget, availability and cost. Other possibilities might be the importance of cleaning between teeth was apparently less well understood or underestimated by patients.^11^ It is always the responsibility of dentists to emphasize on the application of interdental aid as a preventive tool. Dental flossing along with other methods of oral hygiene practices should be taught at the school level, to be incorporated as a routine oral hygiene measure.

A case of interproximal bone loss, indicating a type of factitial injury resulted by chronic trauma from improper flossing was reported by Walters, et al.^12^ He also emphasized not to overlook the patient's flossing technique when trying to determine the etiology of unusual cementoenamel junction lesions - particularly those with an interproximal location.^12^ Thus, it is of utmost importance to practically demonstrate the flossing technique judiciously to patients rather than just prescribing only.

A study by Nakamura, et al. reported that dentists who were demonstrated dental flossing procedure at the dental schools by their teachers recommended dental floss more frequently among their patients, compared with those who did not see demonstrations of flossing.^13^

During assessment of attitude towards dental floss in the current study, 84 (59.2%) dentists strongly agreed that dental floss is an essential oral hygiene aid along with the toothbrush and 61 (43.0%) strongly agreed that routine dental flossing is necessary.

Higher number of respondents agree that dental floss is not as well marketed as other oral hygiene aids in Nepal. Sharda, et al. reported that print and mass media always influence the choice of oral hygiene aids in India, which holds true even in our country.^14^ So, in the days ahead, adequate importance should be given even to dental floss from all sectors; mass media essentially need to highlight the importance of such measures at a community level.

Eighty-seven (61.3%) of dentists agreed about the lack of awareness regarding dental floss among people in Nepal and their role in creating awareness and motivating people to use dental floss regularly. The majority of the dentists responded that dental floss is not given adequate importance in undergraduate and postgraduate dental education, which may have an negative impact on prescription of dental floss. Thus they emphasized the need for dental flossing training education in the dental curriculum. Dental flossing should be taught at the school level, to be incorporated as a routine oral hygiene measure. It is also equally important to educate and motivate dental students to adopt recommended oral self-care (ROSC) procedures, like flossing, themselves, as this in turn help to encourage them to advocate the same to their patients also.^15^

Good adoption of flossing habit by large number of dental and health care providers could be due to their increased academic levels, dental education experience gained from basic dental subjects, preventive courses and clinical training, and moreover their self-awareness and self-motivation.^16^ In contradictory to the findings of these study Bala, et al. documented that 98.2% of the participants had no idea of dental floss and use of dental floss was reported as only 2.4%.^17^

Likewise, in a study conducted among patient attending dental institution in Bangalore, India with an aim to assess their preventive oral health knowledge, practice and behavior, very low percentage of 7.6% of the subjects were found to use dental floss.^18^ The minimal use can be attributed to the lack of oral health education and awareness in rural set ups. This highlights the rapid need for educating and motivating the public to use this efficient method for oral health care. The findings of our study showed that there is a need to sensitize the adults in rural areas by organizing community based outreach sessions so that they can prioritize oral health and inculcate better oral health practices in their daily regimens and that of their children at an early age.

Nakamura, et al. reported 23.4% of Japanese dentists using floss once a day after brushing.^13^ A study was conducted among various health care professionals in the United States in 2002 where 56.3% of dentists of the United States flossed once a day after brushing their teeth. It was taken note that persons flossing less than once a day were as likely to have periodontitis as those who flossed daily after controlling for the profession, age, gender, smoking, diabetes, coronary heart disease, history of periodontal surgery, and the number of teeth present.^19^

On analysing the results of one study, the observed benefit of flossing did not show a dose response, recommending that flossing two to four times a week combined with brushing may be sufficient to guarantee against periodontitis. These findings can be possibly hypothesized as the removal of interproximal plaque on a daily basis may not be necessary to mitigate disease initiation and/or progression. The composition of the plaque microflora changes over time due to a series of complex interactions, named microbial succession. In order to halt the microbial succession process that leads to a more mature plaque which is widely understood to be associated with inflammation and disease initiation, mechanical disruption of the inter-proximal plaque as infrequent as every few days may be sufficient.^20^

A study conducted among 44 dental students of School of Dentistry, Kerman, Iran, came up with a conclusion that utilization of dental floss in every other day is sufficient to preserve the gingival health if a person with normal periodontal tissues uses the toothbrush and dental floss properly.^21^ On further analysis, more number of respondents were found to use dental floss after brushing their teeth in our study. However, the findings from study by Mazhari, et al .showed that flossing followed by brushing is preferred to brushing and flossing in order to increase fluoride concentration and diminish interdental plaque and in interdental plaque. They attributed flossing initially to loosen bacteria and debris from between the teeth, brushing afterward (when the mouth is rinsed with water) further clears these particles from oral cavity.^22^

The study can be more generalized by involving all the dentists and general population. Practical demonstration of dental flossing by the participants can be incorporated rather than just a questionnaire survey only. Dental examination of participants may add further to assess link between the knowledge, attitudes and practices regarding dental floss and clinical outcomes. Thus, more detailed studies probing in depth about the knowledge, attitude and practice and assessing the clinical aspects can be explored further.

The information obtained will provide a baseline data which could be used by policy makers to develop strategies aimed at improving the oral hygiene of the public.

## CONCLUSIONS

This study presented an integral overview and provided the baseline information of the dental floss-related attitude among dentists in Chitwan. Based on results of this study, the mean attitude score regarding dental floss among dentists in our study is similar to the findings of other studies. The majority of dentists seemed to have a positive attitude towards dental floss, but further improvements can be encouraged and facilitated which could help in influencing the oral health status of a general population in a positive manner. Further similar studies can be conducted considering dental professionals as well as a general population on a larger scale.
